# 

**Published:** 1916-09-16

**Authors:** 


The Military Cross has been awarded to Lieutenant P. M. Turnbull, R.A.M.C., the senior assistant medical officer at Tooting Asylums, for conspicuous gallantry in attending to wounded under heavy fire on several successive days. Sergeant T. H. Simmons, Worcester Regiment, an attendant at Leavesden Asylum, has been awarded the Military Medal for conspicuous gallantry and good work at La Boiselle, and in helping wounded men in very exposed positions into comparative safety. Sergeant Simmons has been wounded since his brave deeds brought him recognition, but is progressing favourably. The Metropolitan Asylums Board has passed resolutions expressing the Board's high appreciation of Lieutenant Turnbull and Sergeant Simmons's devotion to duty.

### HOSPITAL AND INSTITUTIONAL RESULTS IN 1915

**Orange Memorial Hospital, Orange, New Jersey.** The hospital receives pay patients to private rooms, to semi-private rooms, and to wards, also part-pay patients and entirely free patients. The total admitted in 1915 was 2,274, and the free patients represent 40 per cent. of this number. There was a "loss on hospital operations" in 1915 of about £12,000, but the general fund from which this was taken stands at over £28,000.

**Preston and County of Lancaster Queen Victoria Infirmary.**—In-patients have increased in number, and out-patients have decreased. Total income, £8,528; total expenditure, £8,140. There seems to be no reason why the accounts should not be kept and presented in accordance with the Uniform System.

**West Sussex County Asylum, Chichester.**—The 677 patients lately in the Graylingwell Asylum, on that building becoming a war hospital, have been distributed amongst ten other institutions, and the report of the Visiting Committee therefore refers to work done in other asylums which are visited by members of the committee.

**Joint Counties Mental Hospital, Carmarthen.**—The number of inmates has been augmented by the admission of patients from Cardiff Mental Hospital, which is being used as a military hospital. About one-third of the attendant staff have joined the Colours. There are forty-six private patients paying from 11s. to 32s. towards their cost of maintenance.

### HOME OF REST FOR ITALIAN SOLDIERS.

As the disabled Belgian soldiers who have been under the care of the Wounded Allies Relief Committee (of Sardinia House, Kingsway, W.C.) at the Home for Disabled Belgian Soldiers, 45 Courtfield Gardens, Kensington, are now able to support themselves through the help of the committee in providing them with artificial limbs and in training them in various industries, this particular Home has been closed, and the furniture, bedding, etc., are about to be presented by the committee to the Italian Red Cross for the benefit of a new "Home of Rest" for sick and wounded Italian soldiers. The Home, for which beds and other furniture are needed, is situated among the hills behind Cormons.

Dr. J. T. Leon, of Southsea, who died on March 30 leaving an estate of the gross value of £37,878, bequeathed £500 to the Royal Portsmouth Hospital, £300 to the Eye and Ear Infirmary, Portsmouth, £100 to the Portsmouth Charity Organisation Society, and £50 to the Victoria Association for Nursing the Sick Poor.

### The Book Market.

Edward Arnold: "The Treatment of Diseases of the Skin." By W. K. Sibley. 6s. net.

Sampson Low, Marston and Co.: "Low's Handbook to the Charities of London." 1916. 1s. net.

### Institutional and Public Reports.

Report on the Health of Portsmouth for the Year 1915. By A. Mearns Fraser, M.D., Medical Officer of Health; including the Report of the Medical Superintendent, Milton Hospital, and of the Public Analyst.

Forty-fifth Annual Report of the Local Government Board, 1915-1916. Part II.: Housing and Town Planning.

### Papers, Pamphlets, etc.

A B C Guide to Seaside and Country Hotels, etc., on the Great Northern Railway and East Coast. Price 1d. (Walter Hill, 67 and 69 Southampton Row, W.C.)

Diphtheritic Whitlow. By J. D. Rolleston, M.D. Reprinted from the *British Journal of Dermatology*.

### Matrons' and Sisters' Appointments.

Halifax, Bermerside Home and Schools.—Miss Elizabeth J. Colley has been appointed matron. She was trained at Towneley Hospital, Bolton, and has since been temporary matron of the Bradford Childrens' Hospital.

Clifton Isolation Hospital, Brighouse.—Miss Florence Elizabeth Whitehouse has been appointed nurse matron. She was trained at Croydon Infirmary, and has since been charge nurse at Brighton Sanatorium, assistant matron and matron of Beacon Hill Infectious Hospital, Faversham, and matron of Bromley Sanatorium, Bury Dispensary and Hospital, and Yarnfield Isolation Hospital, Stone.

Hackney Union Infirmary.—Miss Martha Blanchard has been appointed sister. She was trained at the Ecclesall Bierlow Union, Sheffield, where she was later ward sister, and has since been health visitor at St. Helens.

West Kent Hospital, Maidstone.—Miss Anna Cosgrove has been appointed night sister. She was trained at the West Kent Hospital, and has since done private nursing in London and Maidstone.

**NOTE.—Particulars of Appointments for insertion in this column will be welcomed.**

### Passing Events and Topics.

### An Interesting Gift.

The secretary of Ancoats Hospital, Manchester, has received an anonymous gift of £10 in appreciation of the services rendered in Gallipoli by one of the honorary surgeons to the hospital.

### Extension to Scottish War Hospitals.

A new auxiliary hospital, Hillhead House, Glasgow, placed at the disposal of the Glasgow Red Cross Committee by the trustees of the late Mr. Wilson, has been equipped and attached to Springburn and Woodside Hospitals.

### The After-Care of Consumptives.

Committees charged with the after-care of consumptives are to be established in the Derbyshire towns of Belper, Heanor, Long Eaton, and Wirksworth. At present 102 persons are undergoing treatment at the Walton Sanatorium, Chesterfield.

### The Treasury and Welsh Insurance Commissioners.

Subject to the scheme being approved by the Welsh Insurance Commissioners, the Treasury has consented to the payment of travelling expenses and subsistence allowances to members of the Welsh National Memorial Association for the Treatment of Consumption.

### Memorial Balcony at Salisbury Infirmary.

On August 24, at Salisbury Infirmary, the Rev. A. C. Radcliffe, Rector of Rockbourne, opened a covered balcony which he and Mrs. Radcliffe have presented to Radnor Ward in memory of their son, 2nd Lieutenant W. Y. Radcliffe, who died after being wounded in the struggle for Chunak Bair, Gallipoli, in August 1915.

### Healthy Finances at Stockport Infirmary.

The efforts made to obtain the support for Stockport Infirmary of the munition workers of the district is largely augmenting the contributions from employees. Two flag days and an open-air musical festival have resulted in over £800 being added to the infirmary's funds, as against £280 in 1915. All sources of income have been increased. Forty-eight of the 124 beds are allotted to sick and wounded soldiers.

### Personalia.

Miss Susie E. Hill, M.D., B.S. (Lond.), has been appointed junior assistant medical officer of the St. Pancras House and junior assistant medical superintendent of the St. Pancras South Infirmary, to fill the vacancy caused by Dr. Mabel Bishopp being called up for military work.

Captain Joseph Wilkie Scott, M.D., R.A.M.C., of Nottingham, who, as announced in the *London Gazette*, has been awarded the Military Cross, was successively house physician at the Glasgow Royal Infirmary, the Monkwearmouth Hospital, Sunderland, and the Nottingham General Hospital.

RATES OF SUBSCRIPTION: Twelve months, 6/6; Six months, 4/-; Three months, 2/-, post free to any address in the United Kingdom. Abroad, Twelve months, 8/8; Six months, 5/-; Three months, 2/6, post free. THE HOSPITAL "Index" to Volume LIX., October 1915 to March 1916, is now ready, price 1½d. per copy, post free. Address The Manager, "THE HOSPITAL," The Hospital Building, 28/29 Southampton Street, Strand, London, W.C.

